# Mitochondria Transfer in Mesenchymal Stem Cells: Unraveling the Mechanism and Therapeutic Potential

**DOI:** 10.2174/011574888X362739250416153254

**Published:** 2025-04-25

**Authors:** Jingyi Chen, Zhilang Xie, Huayin Zhou, Yingxin Ou, Wenwen Tan, Aizhen Zhang, Yuying Li, Xingliang Fan

**Affiliations:** 1 Institute of Biological and Food Engineering, Guangdong University of Education, 351 Xingang Middle Road, Guangzhou, 510303, P.R. China

**Keywords:** Mesenchymal stem cells, mitochondrial transfer, immunomodulation, oxidative stress, therapeutic potential, tunneling nanotubes, extracellular vesicles, cell apoptosis

## Abstract

Mesenchymal stem cells (MSCs) hold transformative potential in translational medicine due to their versatile differentiation abilities and regenerative properties. Notably, MSCs can transfer mitochondria to unrelated cells through intercellular mitochondrial transfer, offering a groundbreaking approach to halting the progression of mitochondrial diseases and restoring function to cells compromised by mitochondrial dysfunction. Although MSC mitochondrial transfer has demonstrated significant therapeutic promise across a range of diseases, its application in clinical settings remains largely unexplored. This review delves into the novel mechanisms by which MSCs execute mitochondrial transfer, highlighting its profound impact on cellular metabolism, immune modulation, and tissue regeneration. We provide an in-depth analysis of the therapeutic potential of MSC mitochondrial transfer, particularly in treating mitochondrial dysfunction-related diseases and advancing tissue repair strategies. Additionally, we propose innovative considerations for optimizing MSC mitochondrial transfer in clinical trials, emphasizing its potential to reshape the landscape of regenerative medicine and therapeutic interventions.

## 1 INTRODUCTION

Mesenchymal stem cells (MSCs) are a category of adult multipotent progenitor cells [1], belonging to stromal cells characterized by their ability to self-renew and differentiate into multiple cell lineages [2]. The minimum criteria definition of human MSCs was established by the International Society for Cell Therapy in 2006, which includes their ability to adhere to plastic surfaces, expression of specific markers such as CD105, CD73, and CD90, and the absence of the ability to express certain molecules such as CD14, CD79a, and HLA-DR. Additionally, they can differentiate into osteoblasts, chondrocytes, and adipocytes [3]. MSCs originate from a diverse array of sources and can be extracted from various tissues and organs of the human body. Presently, MSCs are retrievable from bone marrow, fat, umbilical cord blood, human menstrual blood, and the human endometrium [4-8]. The role of MSCs is increasingly prominent in both biological and clinical therapeutic contexts. These cells possess the capacity to differentiate into multiple cell lineages and facilitate tissue repair [2]. Concurrently, they influence cells in the immune system through cytokine secretion, such as natural killer cells (NK), dendritic cells (DCs), B lymphocytes,T lymphocytes, *etc*. Following administration, MSCs are capable of homing to sites of inflammation [9], thereby exerting their immune regulation effects. In addition, MSCs can export their mitochondria for delivery to other cell types, by which to regulate the physiology and function of other tissues and organs in diseases [10].

Mitochondria play a crucial role in the vital movement of eukaryotic cells. They not only supply cellular energy by producing significant amounts of adenosine triphosphate (ATP) through the tricarboxylic acid cycle and oxidative phosphorylation but are also intricately involved in cell responses and the maintenance of homeostasis [11]. Cells harboring damaged mitochondria may encounter a series of issues and may even face demise. Replacing damaged mitochondria can restore normal cellular function [11]. MSCs achieve their therapeutic effects in immunity and regenerative repair through various mechanisms and the release of diverse factors. Among the therapeutic effects of MSCs in regeneration and immunomodulation, mitochondrial transfer has emerged as a swifter and more effective method. Through mitochondrial transfer, damaged or ageing cells can receive mitochondria transfer from healthy cells, facilitating regenerative repair [12, 13]. Upon receiving mitochondria released from damaged cells, MSCs facilitate mitochondrial transfer through mechanisms such as tunnelling nanotubes (TNTs) and extracellular vesicles (EVs). This process aims to replace dysregulated mitochondria in the damaged cells, maintain mitochondrial homeostasis, and respond to reactive oxygen species (ROS) and damage-associated molecular patterns (DAMPs) while also regulating cell metabolism and eliciting immune responses [12, 13]. Mitochondrial transfer plays a pivotal role in stem cell therapy, with a focus on addressing conditions such as cardiovascular diseases, neurological disorders, respiratory diseases, immunodeficiency, venereal diseases, tumors, *etc*. Recent research emphasizes the potential of MSC mitochondrial transfer in halting the spread of mitochondrial diseases and restoring functionality to cells with damaged mitochondria [14, 15].

Despite the significant potential demonstrated by MSC mitochondrial transfer in treating various diseases, its adoption as a therapeutic approach has not been applied in real clinics yet. In this review, we compile the clarified mechanisms behind mitochondrial transfer in MSCs, analyzing the structural aspects, execution, and importance for cellular function and life processes. Furthermore, we outline the therapeutic application of MSC mitochondrial transfer across different diseases, analyzing the inherent challenges and obstacles associated with this process. We also gather emerging technologies for mitochondrial transfer, emphasizing its considerable potential in medical treatment. Finally, we assess the current state of MSC mitochondrial transfer and explore its prospects, highlighting the challenges to be addressed for its continued advancement. To ensure a rigorous and transparent review process, a comprehensive literature search was conducted across PubMed/MEDLINE, Scopus, and Web of Science, using keywords such as “mesenchymal stromal cells (MSCs)”, “mitochondria transfer”, “mitochondrial donation”, “oxidative phosphorylation”, “reactive oxygen species (ROS)” and disease-specific terms, with articles published up to September 2024 in English included. Studies were screened against inclusion criteria focusing on primary research investigating mitochondrial transfer in MSCs, functional outcomes (*e.g*., ATP, ROS modulation), and therapeutic applications, while excluding non-peer-reviewed works, or studies lacking direct mitochondrial transfer evidence. Data extraction covered study design, functional/therapeutic outcomes, and transfer mechanisms (*e.g*., tunneling nanotubes) for animal and human studies, respectively. Findings were synthesized qualitatively into mechanisms, functional implications, and therapeutic applications of MSC mitochondria transfer, and with cross-validation of key results (*e.g*., ATP restoration in cardiomyopathy). Quantitative meta-analysis was omitted due to methodological heterogeneity, ensuring transparency and alignment with the evidence presented.

## 2 MECHANISMS OF MITOCHONDRIA TRANSFER IN MSCs

### 2.1 Tunneling Nanotubes

TNTs are nanotubes larger than 0.7 microns in diameter composed of F-actin and microtubules, facilitating the transport of vesicles and mitochondria [16]. The target apoptotic cells could emit “find-me” signals during the early apoptosis phase. MSCs detect signal gradients and extend membrane protrusions toward injured target cells, establishing connections between them. This process involves the recognition of specific signals, such as the phosphatidylserine domain on damaged cells, leading to the formation of TNT structures with the target cells. Signals emitted by damaged target cells, such as DAMPs and ROS released during oxidative stress and inflammation, activate Miro-1 and Miro-2 proteins (two Rho-GTPases) in MSCs, which interact with auxiliary proteins like TRAK 1, TRAK 2, Myo10, and Myo19, recruiting kinesin to bind to mitochondria, forming an adaptor-motor protein complex. This complex facilitates the unidirectional transport of functional mitochondria along the TNT structures to damaged cells, rescuing them from apoptosis [12, 17-20]. In addition, Yao, Y. *et al*. observed the presence of connexin 43 (CX43) within the TNTs formed between human induced pluripotent stem cell-derived mesenchymal stem cells (iPSC-MSCs) and BEAS-2B cells. They further demonstrated that silencing CX34 in iPSC-MSCs led to a reduction in TNT formation between the two cell types, consequently decreasing mitochondrial transfer. These findings suggest that CX43 regulates TNT formation and facilitates MSC mitochondrial transfer [21, 22].

### 2.2 Extracellular Vesicles

Exosomes and microvesicles (MVs) are both types of EVs, ranging in diameter from 30 to 1000 nm. MVs are vesicles formed when mitochondria are encapsulated within MSCs and then directly released from the cell membrane. Additionally, mitochondria-derived vesicles (MDVs), a specialized subset of EVs, bud directly from mitochondrial membranes and selectively package functional mitochondrial components such as proteins, lipids, and mitochondrial DNA (mtDNA). These MDVs play critical roles in mitochondrial quality control and intercellular communication by delivering bioactive cargo to recipient cells [23, 24]. Interestingly, there are differing views on whether MDVs should be defined as innocent bystanders or accomplices in disease. In the review by Mishra S. & Deep G., MDVs transfer functional ATP synthase subunits to ATP-deficient mitochondria, acting as ‘nano-batteries’ to restore energy production, positioning them as innocent bystanders that mitigate disease by repairing mitochondrial dysfunction [25]. Conversely, several studies highlight the dual nature of MDVs: while they protectively remove oxidized cargo under stress, they can also act as accomplices by packaging and spreading harmful proteins (*e.g*., α-synuclein) through extracellular vesicles, exacerbating disease progression [26, 27]. However, it is important to note that MDVs have different physiological significance in various cell types *in vivo*, and their mechanisms of action are not fully understood, making it difficult to generalize their role. Nevertheless, the therapeutic potential of MDVs is widely recognized in the treatment of various diseases. As therapeutic assistants, MDVs can deliver ATP-generating machinery to rejuvenate damaged mitochondria, offering a novel approach for diseases like Parkinson's disease (PD) [28, 29]. Additionally, some scientists define MDVs as therapeutic targets, where modulating their formation (*e.g*., through cannabidiol-induced MDV production) can counteract mitochondrial dysfunction in neurodegeneration or enhance immune responses in infections [30]. These examples demonstrate that MDVs, by addressing mitochondrial dysfunction, serve as a means to treat diseases, undoubtedly holding significant promise in regenerative medicine. They act as a ‘backup battery’, providing energy to mitochondria that have lost normal function, which is crucial for cell survival and tissue repair [25]. Furthermore, studies have shown that MDVs decline in aging and frailty, which means that promoting MDV secretion could restore mitochondrial homeostasis [31]. Fragments of mitochondria or mitochondrial RNA (mtRNA) can be enclosed within multivesicular bodies inside MSCs. Subsequently, the membrane of these multivesicular bodies fuses with the plasma membrane, leading to the release of vesicles as exosomes [23]. The membranes of EVs harbour receptor proteins (Integrin, intercellular adhesion molecule 1, milk fat globule epidermal growth factor 8 protein, *etc*.) that recognize specific proteins (lymphocyte function-associated antigen 1, T cell immunoglobulin domain and mucin domain protein 1, T cell immunoglobulin domain and mucin domain protein 4, *etc*.) on the surface of recipient cells. Upon fusion with the recipient cell's cytoplasmic membrane, the substances within the EVs enter the cytoplasm of the recipient cell to carry out their respective functions [32, 33]. MSCs, which survive hypoxic conditions *in vivo*, experience oxidative stress in hypoxic environments when cultured in atmospheric conditions, triggering mitochondrial autophagy mediated by the Pink1/Parkin signaling pathway. This process leads to the formation of mitochondrial vesicles, expressing various proteins including arrestin domain-containing protein 1, which directs MVs towards the plasma membrane and ultimately separates from MSCs. Upon recognition and phagocytosis by macrophages, the mitochondria in MVs will fuse and contribute to macrophage bioenergetics and enhance MSC survival [34, 35].

### 2.3 Gap Junctions

It was found that connexins can assemble into gap junction channels (GJCs) between MSCs and damaged cells, facilitating the transfer of mitochondria [23, 36]. The varied origins and culturing environments of MSCs can result in the expression of diverse connexins, which in turn mediate the formation of gap junctions [22, 37]. Islam, M.N., *et al*. found that bone marrow-derived mesenchymal stem cells (BM-MSCs) can transfer mitochondria to alveolar epithelial cells through gap junctions, which was proposed as a strategy to counteract acute lung injury (ALI). When foreign substances intrude into the alveoli, causing mitochondrial dysfunction in alveolar epithelial cells, intracellular CX43 expression increases. Bone marrow-derived mesenchymal stem cells (BM-MSCs) migrate into the alveoli, adhering to areas with elevated CX43 expression. Subsequently, GJCs form between BM-MSCs and alveolar epithelial cells, facilitating Ca2^+^ communication. Additionally, BM-MSCs generate nanotubes and microvesicles containing mitochondria, which are engulfed by epithelial cells, boosting ATP levels in the alveolar epithelium [36]. Aside from the homotypic GJCs facilitated by CX43, Li, H. *et al*. discovered that motoneurons exclusively express CX32 while BM-MSCs express CX43. Interestingly, both cell types establish a heterotypic GJC containing CX32 and CX43 to facilitate mitochondrial transfer from BM-MSCs to oxygen-glucose deprivation-injured motoneurons, indicating the potential of heterotypic GJC to mediate mitochondrial transfer [38]. These findings suggest that CX43-based GJCs may directly or indirectly regulate mitochondrial transfer together with TNTs. However, there is currently no relevant study elucidating the specific role of CX43-based GJC formation on TNT and the underlying mechanisms governing mitochondrial transfer.

In addition to CX43, different sources and culture environments of MSCs can lead to the expression of different connexin proteins, which, in turn, mediate the formation of gap junctions. Sinclair, K.A. *et al*. found that MSC isolated from bronchoalveolar lavage fluid of lung transplant recipients blocked the formation of CX43 gap junctions. Despite this, the transfer of mitochondria from MSCs to bronchial epithelial cells remains unaffected, suggesting that MSCs can utilize non-CX43 gap junctions for cytoplasmic mitochondrial transfer to bronchial epithelial cells. It is hypothesized that an appropriate alternative may be CX26, which is expressed by basal cells [37].

## 3 FUNCTIONAL IMPLICATIONS OF MITOCHONDRIA **TRANSFER IN MSCs**

The functional implications of MSC mitochondrial transfer involve various cellular processes and have significant implications for tissue homeostasis, repair, and disease modulation. MSC mitochondrial transfer has a profound impact on cellular metabolism and bioenergetics, enhancing ATP production, regulating oxidative phosphorylation, reducing ROS levels, maintaining mitochondrial membrane potential, and modulating cellular respiration. These effects contribute to the overall functional implication of MSC mitochondrial transfer in cellular metabolism and function (Table **1**).

### 3.1 Impact on Cellular Metabolism and Bioenergetics

#### 3.1.1 Enhancement of Oxidative Phosphorylation

**Fig. (1):**
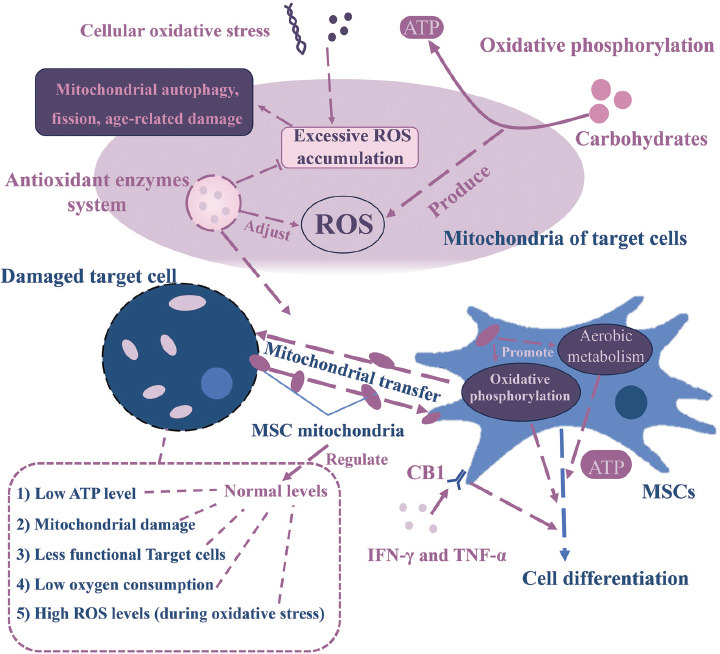
Functional implications of mesenchymal stem cell (MSC) mitochondria transfer. Mitochondria produce ATP through oxidative phosphorylation, breaking down carbohydrates, in which by-products such as reactive oxygen species (ROS) are produced. In damaged cells, the mitochondrial function is usually damaged, which leads to the decrease of intracellular ATP level, cell function and oxygen consumption, thus affecting the energy metabolism of cells. Under conditions that induce oxidative stress in cells, ROS levels increase significantly, triggering mitochondrial dynamics and leading to oxidative damage in cells, such as Mitochondrial autophagy, fission, age-related damage. Transferring mitochondria from MSCs to damaged recipient cells can restore their mitochondrial function and energy metabolism. The antioxidant oxidase system in mitochondria can help mitochondria regulate their ROS level, promote the transfer of healthy mitochondria to damaged cells. Notably, mitochondrial transmission of MSCs to recipient cells is bidirectional. The differentiation ability of MSCs is closely related to mitochondrial biogenesis, which plays a key role in MSC differentiation through changes in glycolysis and oxidative phosphorylation. The extracellular environment also plays a significant role in the differentiation of MSCs.

Mitochondria serve as biological powerhouses, converting nutrients into energy to fuel cellular activities [39]. They are pivotal in oxidative phosphorylation and the generation of ATP, which is essential for normal cellular functions and bodily movements. This process primarily involves breaking down carbohydrates through oxidative phosphorylation to produce ATP [40], thus maintaining cellular energy balance within metabolic pathways (Fig. **1**). Oxidative phosphorylation is crucial for preserving cellular energy equilibrium within the metabolic pathways of mitochondria. The oxidative phosphorylation system comprises the FOF1-ATP synthase and four respiratory complexes within the inner mitochondrial membrane [41].

Additionally, fatty acid oxidation, a multifaceted biological process [42], involves the complete oxidation of fatty acids into carbon dioxide and water through pathways such as β-oxidation, the tricarboxylic acid (TCA) cycle, and the electron transport chain (ETC). These processes also participate in the oxidative metabolism of proteins and carbohydrates [43]. Furthermore, in redox processes, respiratory complex I and respiratory complex III modulate the equilibrium of signaling pathways engaged in cell growth and stress response by generating ROS [40].

The pathogenesis of nearly all chronic diseases involves oxidative phosphorylation dysfunction. Mitochondria possess the ability to infiltrate host cells, and heightened mitochondrial enzyme activity correlates with improved oxidative phosphorylation. MSCs exhibit responsiveness to damaged or inflamed tissues by transferring mitochondria to affected cells and immune cells. This transfer boosts ATP production and oxygen consumption, thereby sustaining cellular bioenergy levels and reinstating cellular functionality [39, 44, 45]. As shown in Fig. (**1**), the transmission of functional mitochondria from MSCs to damaged recipient cells, such as in lung epithelium, neurons, and myocardium, can rejuvenate their mitochondrial function and energy metabolism [46]. Studies have demonstrated an increase in ATP levels in airway epithelial cells following the mitochondrial transfer from bone marrow-derived MSCs (BM-MSCs) to lung epithelial cells in models of airway injury [47]. Furthermore, animals subjected to mitochondrial transfer from MSCs exhibited elevated citrate synthase activity and a higher maximal oxygen consumption rate [44]. By receiving mitochondria from MSCs, immunocompetent cells can obtain signals aiding in the restoration of immune homeostasis. In the case of macrophages, the acceptance of mitochondria transferred by MSCs augments their phagocytic capacity and ATP turnover rate [45]. The mechanism of mitochondrial oxidative phosphorylation plays a significant role in inducing M2-type macrophages *via* IL-4 signaling. Morrison, T.J., *et al*. found that the conveyance of functional mitochondria through MSC-derived EVs promotes oxidative phosphorylation, thus fostering phagocytosis by human macrophages and dampening the secretion of pro-inflammatory cytokines [48]. Similarly, these mechanisms have implications for malignant tumors. The receipt of mitochondria and/or mitochondrial DNA by the transfer of cancer cells can elevate their mitochondrial content, amplify oxidative phosphorylation, increase ATP production, and promote the proliferation and metastasis of cancer cells [49, 50]. Additionally, transferred MSC mitochondria possess the potential to be internalized by cancer cells and utilized to enhance their bioenergetics [51].

#### 3.1.2 Regulation of ROS

ROS is an oxygen-containing compound, categorized into types including superoxide (O^2-^), hydrogen peroxide (H_2_O_2_), hydroxyl radical (OH^-^), and lipid hydroperoxide [52]. ROS exhibit potent biological activity and readily interact with organic substrates like proteins, lipids, nucleic acids, and sugars, contributing to the generation of oxygen free radicals in biochemical reactions [13]. ROS, as a by-product of mitochondrial oxidative phosphorylation, plays a positive role in maintaining the biological activity of MSCs at normal levels (Fig. **1**). They can enhance cell signaling and transcriptional regulation with surrounding tissues [44]. Typically, ROS levels are regulated through scavenging mechanisms within mitochondria, and cellular homeostasis is maintained *via* the metabolism of toxic intermediates by the antioxidant enzymes present in cells [12]. SIRT3, a prominent mitochondrial protein deacetylase, instigates metabolic reprogramming and plays a pivotal role in ROS level control by diminishing carbohydrate catabolism (Fig. **1**) [52]. However, under induced conditions like hypoxia-ischemia, mitochondrial DNA deletion, and chemical exposure, which induce cellular oxidative stress, heightened electron leakage from the respiratory chain leads to substantial ROS accumulation within mitochondria, causing a significant rise in ROS levels [53]. Oxidative stress denotes an imbalance between the production of free radicals and the body's capacity to neutralize or counteract their detrimental effects through antioxidant defense mechanisms [54]. Elevated ROS production serves as a stimulant for mitochondrial autophagy [55]. Heightened ROS levels instigate mitochondrial dynamics, resulting in fragmentation *via* autophagy and fission [12]. During periods of stress, cellular regulatory mechanisms such as antioxidant enzymes, responsible for metabolizing toxic intermediates, are unable to maintain mitochondrial homeostasis. This leads to heightened mitochondrial autophagy, downregulation and dysfunction of biogenesis, and exacerbation of aging-related damage [11, 56]. Excessive ROS accumulation induced by stress can result in cellular oxidative damage, encompassing DNA damage, protein oxidative modification, and lipid peroxidation [44, 57]. Simultaneously, it prompts the transfer of healthy mitochondria into cells with impaired mitochondria to regulate surplus ROS production and reinstate mitochondrial function [58, 59].

ROS, AMP/ATP, Ca^2+^, and NAD^+^/NADH ratios within cells experiencing oxidative stress conditions instigate the transmission of retrograde signals, subsequently prompting the transfer of mitochondria from MSCs to recipient cells. Research indicates that following co-cultivation with MSCs, recipient cells exhibit a reduction in mitochondrial ROS levels, a decline in respiratory chain complex activity, regulation of cellular metabolism in injured tissues, and reversal of oxidative stress conditions [12, 49, 60]. Furthermore, mitochondria obtained from BM-MSCs demonstrate the ability to suppress ROS production and enhance the expression of mitochondrial SOD enzyme and Bcl-2 protein. Mitochondrial transfer from BM-MSCs to renal cells *via* intravenous injection suppresses ROS production and modulates mitochondria-associated factors such as Bcl-2, PGC-1α, and BAX. This modulation aims to restore the expression of SGLT2 and the megalin transporter, thereby reinstating renal tubular function [47]. Conversely, mitochondria obtained from platelets hinder the rise in intracellular mitochondrial ROS induced by TGF-β and cisplatin in hDFs [58]. Furthermore, mitochondrial transfer can occur bidirectionally (Fig. **1**). There is evidence of bidirectional mitochondrial transfer between BM-MSCs cells and T-cell acute lymphoblastic leukemia (T-cell ALL) cells. Following chemotherapy, T-cell ALL cells acquire fewer mitochondria from adherent BM-MSCs compared to the mitochondria transferred from T-cell ALL cells to BM-MSCs. This results in reduced mitochondrial ROS in T-cell ALL cells, thereby enhancing chemoresistance [21, 53]. Additionally, mitochondrial exchange takes place between hematopoietic stem and progenitor cells (HSPCs) and MSCs. Mitochondria are transferred more extensively from h HSPCs to MSC cells than from MSCs to HSPCs. This exchange serves to scavenge ROS within the donor hematopoietic progenitor cells and supports metabolic activity and regenerative function in recipient MSCs. Additionally, this process enhances the likelihood of successful hematopoietic transplantation [61]. Under oxidative stress conditions, MSCs derived from various tissues exhibit varying degrees of reduction in mitochondrial ROS and mitochondrial translocation. BM-MSCs and adipose tissue-derived MSCs (AD-MSCs) demonstrate a high level of mitochondrial translocation, along with reduced bioenergetics and lower respiratory capacity compared to dental pulp-derived MSCs (DP-MSCs) and Wharton's jelly-derived MSCs (WJ-MSCs) [51].

### 3.2 Modulation of Stem Cell Properties and Functions

#### 3.2.1 Differentiation Potential and Lineage Commitment

Mitochondria also play a regulatory role in ROS levels, which impacts cell differentiation and contributes to the determination of the direction of differentiation (Fig. **1**). The differentiation capacity of MSCs is closely intertwined with mitochondrial biogenesis, which plays a pivotal role in the differentiation process of MSCs by diminishing glycolysis and augmenting oxidative phosphorylation, thus ensuring sufficient energy production to sustain the metabolic demands of MSCs [62]. Mitochondria and the regulatory factor deacetylase associated with mitochondria play a crucial role in governing the homeostasis of MSCs and their differentiation into mature osteoblast and adipocyte lineages [13, 63]. During the initial phases of MSC differentiation, mitochondrial dynamics undergo alterations, including both fission and fusion, as well as changes in density, activity, morphology, and distribution (Fig. **1**). Notably, during osteogenesis and adipogenesis, an enhancement in mitochondrial morphological elongation and fusion is observed. During chondrogenesis, there's an observed increase in mitochondrial fission and autophagy [56]. A defining characteristic of MSC differentiation is the transition from glycolysis to aerobic metabolism as the primary energy source, commonly referred to as the metabolic shift [64]. Throughout MSC osteogenic differentiation, there is a metabolic shift from glycolysis to oxidative phosphorylation, leading to the upregulation of respiratory enzymes and increased mitochondrial oxidative phosphorylation. This process activates acetylation and β-catenin signaling, ultimately promoting mitochondrial biogenesis and oxidative metabolism (Fig. **1**).

During MSC differentiation, various aspects of mitochondrial morphology and function, including perinuclear arrangement, kinetics, metabolic activity, mass, and size, undergo significant alterations, resulting in enhanced mitochondrial oxidative metabolism [65-67]. These functional regulations and dynamics of mitochondria play a pivotal role in the successful differentiation of MSCs. It has been shown that the reduction in the expression of the mitochondrial enzyme Mtu1 leads to respiratory defects and decreased mitochondrial ATP production in BM-MSCs cultured *in vitro*, ultimately impeding the process of osteogenic differentiation. This effect is associated with the reduced modification of 2-thiouridine by mitochondrial tRNA^Glu^, tRNA^Gln^, and tRNA^Lys^ at wobble uridine [68]. Increased mitochondrial activity serves as a decisive factor in the differentiation of MSCs into adipocytes. During MSC adipogenic differentiation, mitochondria undergo a notable increase in biosynthesis and oxygen consumption. Reducing mitochondrial respiration, either through hypoxia or inhibition of the mitochondrial electron transport chain, will result in a significant hindrance to adipocyte differentiation of MSCs (Fig. **1**) [69]. It is worth noticing that hypoxic conditions effectively impede mitochondrial oxidation, leading to the inhibition of adipogenesis and osteogenesis without affecting cartilage formation [70, 71].

Inflammatory conditions detrimentally affect the osteogenic differentiation capacity of MSCs. However, in such environments, the osteogenic differentiation potential of human BM-MSCs can be augmented by stimulating cannabinoid receptor 1 (CB1) through interferon-gamma (IFN-γ) and tumor necrosis factor-alpha (TNF-α) activation (Fig. **1**). This stimulation effectively restores the energy metabolism function of mitochondria [72]. Moreover, the intercellular transfer of mitochondria can serve as a trigger for stem cell differentiation, reprogramming of differentiated cells, or activation of inflammatory signaling pathways. Human MSCs have been observed to inhibit cardiomyocyte apoptosis or degeneration and reprogram fully differentiated mouse cardiomyocytes into a cardiac progenitor cell-like state through fusion with cardiomyocytes and mitochondrial transfer [23, 47, 69].

#### 3.2.2 Proliferation and Self-renewal Capacity

MSCs maintain their multipotency through self-renewal and can also migrate to the site of injury to continuously provide daughter cells for tissue regeneration and replenish mature cells, thereby maintaining homeostasis throughout the life cycle of the organism [73-75]. Signaling molecules such as FoxOs, Nrf2, p38, and p53 are involved in the self-renewal process of MSCs [76]. After MSCs are transferred and implanted into different tissues, they will rapidly proliferate and undergo differentiation processes, transforming their phenotype into tissue-specific cells [77]. Accumulated mitochondrial damage during cell division may eventually compromise the self-renewal capacity of stem cells. However, the physiological upregulation of ROS is a necessary condition for MSC proliferation, and the inhibition of ROS will hinder the self-renewal of MSCs. The antioxidant enzyme cofactor, ascorbic acid, inhibits the transcription of HIF-1α by activating HIF-1α hydroxylase and increases ROS levels, which in turn promotes BM-MSC proliferation [78]. In addition, the impairment of the mitophagy function also contributes to the generation of mitochondrial ROS, and a moderate hypoxic environment also enhances the proliferation of MSCs [74, 79]. In the process of growth, survival, and mobilization of MSCs, the PI3K/AKT signaling pathway plays a key role. As an upstream activator of PI3K/AKT, Exendin-4 (Ex-4), an antidiabetic drug derived from the saliva of Gila venomous lizards, can be used to enhance the proliferation of MSCs in a dose- and time-dependent manner. Three major intracellular signaling pathways, PI3K/AKT, cAMP/PKA, and Mitogen-activated protein kinase, are activated when Ex-4 is applied to the cells, and the number and lifespan of MSC cells in the cell cycle synthesis (S phase) are significantly increased [80, 81]. Leukaemia inhibitory factor (LIF), a natural multifunctional cytokine in the body, can also promote the self-renewal and differentiation of BM-MSCs by enhancing the activity of the PI3K/AKT signaling pathway to avoid the impact of oxidative stress. In the hypoxic environment of the bone defect area, LIF was activated, and the PI3K/AKT downstream signaling pathway was activated accordingly, thus inhibiting oxidative stress, protecting the self-renewal and survival of BM-MSCs, improving the inhibitory effect of the hypoxic microenvironment on bone growth, and promoting osteogenic differentiation [82].

It was found that upon the transfer of mitochondria from vascular smooth muscle cells to MSCs, direct interaction between MSCs and vascular smooth muscle cells stimulates MSC proliferation. [83]. Through the transfer of healthy mitochondria, recipient MSCs acquire additional mitochondria, leading to heightened aerobic metabolism, such as increased oxidative phosphorylation. This significantly promotes the proliferation, osteogenesis, and migration of MSCs *in vitro*, thereby advancing the healing process of bone defects mediated by MSCs [67]. Direct transplantation of exogenous mitochondria derived from mesenchymal stem cells demonstrates remarkable capabilities in inducing tube formation, enhancing ATP content, stem cell factor (SCF) secretion, and cell proliferation in human umbilical vein endothelial cells (HUVECs). In terms of its mechanism of action, the administered MSC mitochondrion mitigates oxidative stress-induced endothelial senescence by activating the extracellular signal-regulated kinase pathway [84]. The transfer of MSC mitochondria into HUVECs through TNT channels promotes HUVEC proliferation and restores their capacity to form capillaries and migrate across membranes [85]. The regeneration of retinal ganglion cells and corneal epithelial cells relies on the exchange of mitochondria between cells, with MSCs serving as common mitochondrial donors for various eye cells. However, mitochondrial transfer is seldom observed between retinal pigment epithelium (RPE) cells, intergroup corneal endothelial cells, or between RPE cells and intragroup photoreceptors [86].

## 4 THERAPEUTIC APPLICATIONS OF MSC MITOCHONDRIAL TRANSFER

**Fig. (2):**
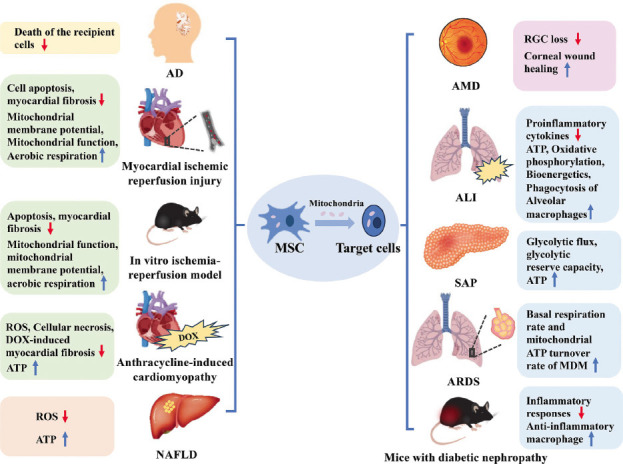
Therapeutic applications of mesenchymal stem cell (MSC) mitochondria transfer. In Alzheimer's disease (AD), human umbilical cord derived MSCs could transfer healthy mitochondria *via* EVs to inhibit cell death of the recipient cells, by which to increase mitochondrial membrane potential, restore mitochondrial function, enhance aerobic respiration, reduce apoptosis and mitigate myocardial fibrosis. In anthracycline-induced cardiomyopathy, MSC mitochondrial transfer increased ATP levels, decreased reactive oxygen species (ROS) production, reduced cellular necrosis and attenuated Adriamycin (DOX)-induced myocardial fibrosis. In non-alcoholic fatty liver disease (NAFLD), MSC mitochondrial transfer enhanced ATP production and reduced ROS levels. In age-related macular degeneration (AMD), MSC mitochondrial transfer accelerated corneal wound healing and prevented retinal ganglion cell (RGC) loss. In acute lung injury (ALI), MSC mitochondrial transfer increased the integrity of the pulmonary microvascular endothelial cell (PMVEC) barrier and ATP concentration, enhanced oxidative phosphorylation and phagocytosis of alveolar macrophages, restored bioenergetics and dampened the secretion of pro-inflammatory cytokines. In severe acute pancreatitis (SAP), MSC transfer mitochondria to pancreatic acinar cells through EVs, which not only up-regulated glycolytic flux, enhanced the glycolytic reserve capacity of pancreatic acinar cells and produced more ATP to resist inflammation. In acute respiratory distress syndrome (ARDS), MSC mitochondrial transfer enhanced basal respiration rate and mitochondrial ATP turnover rate of monocyte‐derived macrophages. In mice with diabetic nephropathy, MSC mitochondrial transfer prompted an anti-inflammatory macrophage and suppressed inflammatory responses.

The therapeutic application of MSC mitochondrial transfer is wide-ranging and holds significant promise for addressing a variety of medical conditions and diseases according to preclinical results. From neurological disorders to cardiovascular diseases and metabolic disorders, MSC mitochondrial transfer holds promise for improving treatment outcomes and enhancing tissue regeneration (Fig. **2**). So far, several studies have investigated the therapeutic functions of MSC mitochondrial transfer in different diseases (Table **2**).

### 4.1 Mitochondrial Dysfunction-related Diseases

#### 4.1.1 Neurodegenerative Disorders

Neurodegenerative diseases, including PD, Alzheimer's disease (AD), amyotrophic lateral sclerosis (ALS) and other diseases, are a group of chronic progressive neurological disorders characterized by neuronal degeneration or apoptosis or gradual deterioration of neurons and glial cells in the brain/spinal cord, with marked decline or even loss of memory, motor, and brain-specific cognitive functions being the hallmarks of neurodegenerative diseases [87, 88]. The brain is one of the most oxygen-consuming organs in the animal body and relies on mitochondria for energy for nerve formation and transmission. Despite the diverse origins and clinical presentations of neurodegenerative diseases, studies suggest that mitochondrial damage and dysfunction, leading to increased ROS formation, dysregulate Ca^2+^ homeostasis and contribute to neuroinflammatory symptoms damaging the central nervous system. These factors are identified as the primary etiological factors of neurodegenerative disorders [89-92]. By utilizing the self-renewal and differentiation capacities of MSCs, mitochondrial transfer or transplantation replaces damaged mitochondria with those from MSCs, allowing both damaged cells and organelles (mainly mitochondria) to regain normal function. This highlights MSC mitochondrial transfer or transplantation as a promising therapeutic approach [93].

AD is one of the most common neurodegenerative diseases in the world. Data showed that under normal conditions, microglia function to eliminate and degrade extracellularly generated amyloid β protein (Aβ), allowing the clearance of Aβ plaque lysosomes [91, 94]. In contrast, in AD pathology, the accumulation of substances (Aβ aggregates and other factors) released by injured neurons impedes microglia activation, which in turn leads to extracellular Aβ accumulation, hyperphosphorylation of Tau proteins, increased oxidative stress, ROS accumulation, and neuronal damage [95]. It has been demonstrated that mitochondrial transfer indeed improves mitochondrial function in the brain in an AD mouse model [96]. Furthermore, in related experiments, results showed that MSCs reduced the hyperphosphorylation of Tau proteins and the accumulation of amyloid plaques, and MSCs prevented the deposition of Aβ plaques and activated microglia [97, 98]. MSCs not only secrete neuroprotective and anti-inflammatory proteins to safeguard neuronal activity but also utilize secreted EVs to transport mitochondria and other organelles to damaged cells, providing essential “new materials” to repair damaged cells, improving the survival environment and, consequently, slowing down the symptoms of AD [93, 99]. In an SH-SY5Y cell model using okadaic acid-treated SH-SY5Y cells, Zhang L. *et al*. found that human umbilical cord-derived MSCs could transfer healthy mitochondria *via* EVs to restore basic energy metabolism and inhibit cell death of the recipient cells and that they restored the normal function of the mitochondria in the AD cell model [99].

PD is the second most prevalent neurodegenerative disease in the world after AD, which is clinically characterized by bradykinesia, resting tremor, and non-motor symptoms [100, 101]. The link between PD and mitochondria was first identified in the 1980s. Since then, numerous *in vivo* and *in vitro* experiments have confirmed that mitochondrial dysfunction significantly contributes to the development of PD [102-107]. In 2009, Venkataramana, N.K., *et al*. transplanted autologous BM-MSCs in PD patients, and the results showed the safety of autologous MSC transplantation [108]. On this basis, Canesi, M. *et al*. administered MSCs *via* cerebral artery injection to patients with PD, observing positive outcomes in 17 subjects during a follow-up visit [109]. However, there is still no direct evidence indicating the role of MSC mitochondrial transfer in PD. Similarly, relevant studies have confirmed that utilizing MSC therapy can indeed reduce the course of ALS, prolong the survival of ALS patients, and reduce the risk of patient death. Syková *et al*. and Siwek T *et al*. conducted similar studies in which patients were given intrathecal injections of autologous BM-MSCs, and both results confirmed the safety and efficiency of MSC interventions in ALS patients [110-112]. Yet, there remains a lack of direct evidence elucidating the role of MSC mitochondrial transfer in ALS.

Overall, experimental and clinical trials related to MSC transplantation in recipients using different sources of MSC in neurodegenerative diseases have been carried out successfully, proving the safety and efficiency of MSC injections and further responding to the great potential of MSC transplantation in clinical treatment. However, research on MSC mitochondrial transfer in neurodegenerative diseases is still in its early stages, with only a few studies investigating it. Nevertheless, MSC mitochondrial transfer may retain significant therapeutic potential for neurodegenerative diseases.

#### 4.1.2 Cardiovascular Diseases

Mitochondrial dysfunction plays a crucial role in the onset and progression of cardiovascular diseases (CVD). This dysfunction is characterized by mitochondrial complex disruption, mitochondrial uncoupling, and cristae remodeling, which lead to impaired energy metabolism and increased ROS accumulation [113]. These disturbances contribute to various cardiovascular conditions, including ischemic cardiomyopathy and anthracycline-induced cardiomyopathy. MSCs show promise in mitochondrial transfer therapy due to their high-quality mitochondria and efficient delivery capacity [114, 115]. Its ability to transfer mitochondria into damaged cells potentially increases mitochondrial numbers and enhances cell function. This mechanism provides a therapeutic pathway for cardiovascular diseases, especially the aforementioned diseases against mitochondrial dysfunction.

In a rat model of ischemic cardiomyopathy, human AD-MSCs were co-cultured with rat cardiomyocytes. This resulted in the transfer of AD-MSC mitochondria into cardiomyocytes, significantly improving cardiac function [116]. Ischemia-reperfusion injury involves the restoration of blood flow after ischemia, which exacerbates tissue damage due to mitochondrial dysfunction and oxidative stress. In an *in vitro* ischemia-reperfusion model, co-culture of H9C2(2-1) cells with MSCs demonstrated that MSCs transferred mitochondria *via* TNTs. This transfer restored mitochondrial function, reduced apoptosis, improved mitochondrial membrane potential, enhanced aerobic respiration, protected the myocardium from oxidative stress, and mitigated myocardial fibrosis [51, 114, 117].

Another type of cardiomyopathy commonly associated with anthracyclines, such as doxorubicin (DOX), is also well treated with MSCs. This disease is mainly due to the accumulation of DOX in cardiomyocytes, which leads to mitochondrial membrane potential collapse and cell apoptosis [118-121]. Mitochondrial transfer has shown potential in reversing DOX-induced mitochondrial damage. In a mouse model of cardiomyopathy, iPSC-MSCs demonstrated increased sensitivity in forming TNTs, transferring and retaining mitochondria in cardiomyocytes, reducing ROS production, improving mitochondrial function, increasing ATP levels, and attenuating DOX-induced myocardial fibrosis [122]. *In vitro* studies with human iPSC-derived cardiomyocytes co-cultured with MSCs after DOX injury revealed that MSCs transferred mitochondria enriched in EVs. This improved cellular physiology by reducing ROS production, enhancing mitochondrial biogenesis and function, increasing ATP production, mitigating DOX injury, and reducing cell apoptosis [118].

In the treatment of such diseases, MSCs have not only made great achievements in preclinical research but also have no significant impact on clinical trials. At present, cell therapy for cardiovascular diseases mainly faces challenges such as cell survival and immune compatibility, and the two therapeutic pathways of MSCs mitochondrial transfer through TNTs give us new hope. Clinical studies have found that by injecting patients with MSCs, MSCs transfer mitochondria to endothelial cells, significantly enhancing their bioenergetics and angiogenesis capabilities [123]. This mitochondrial transfer is essential for the implantation and function of endothelial cells, providing a new strategy for blood vessel regeneration without the need for additional cell types. This will be a new direction in regenerative medicine and provide new hope for the clinical practice of treating cardiovascular diseases.

Although MSCs have shown promising results in the treatment of CVD, many challenges remain. Can TNTs improve the efficiency of mitochondrial transfer? Does MSCs mitochondrial transfer have potential side effects in cardiovascular therapy? These problems remain to be resolved. Overall, while MSC mitochondrial transplantation offers a promising treatment for cardiovascular disease, continued research is needed to address these challenges and realize its full clinical potential.

#### 4.1.3 Metabolic Disorders

Mitochondria serve as the primary site for ATP synthesis, fatty acid β-oxidation, and ROS production. Dysfunction in mitochondria can result in impaired energy metabolism and increased oxidative stress, leading to disturbances in the metabolic state of various substances [124]. Common metabolic disorders such as type II diabetes and non-alcoholic fatty liver disease often arise from such dysfunction [125]. MSCs offer a potential avenue for transferring mitochondria to dysfunctional cells, thereby restoring mitochondrial homeostasis and regulating substance metabolism [114, 115]. Bi, Y. *et al*. observed that administering BM-MSCs to mice with NAFLD attenuated the progression of the disease. They noted mitochondrial transfer between BM-MSCs and fatty liver cells, leading to enhanced ATP production and reduced ROS levels in the liver cells. Blocking mitochondrial transfer hindered these effects, highlighting the potential of MSC-mediated mitochondrial transfer as a promising therapeutic strategy for improving mitochondrial function and restoring damaged liver tissue [126, 127]. It has been reported that mitochondrial transfer derived from BM-MSC is critical for the treatment of diabetic nephropathy (DN). In the DN model, BM-MSCs improve the damage and inhibit the apoptosis of PTECs by transferring mitochondria to proximal tubular epithelial cells (PTECs). By regulating the expression of factors such as Bax, Bcl-2, SOD2, and PGC-1α and effectively inhibiting ROS production, BM-MSC-derived mitochondria can improve endogenous mitochondrial function, thereby inhibiting the apoptosis of PTEC *in vitro*. *In vivo*, BM-MSC transferred mitochondria were able to cross the structurally damaged tubular basement membrane and successfully integrate into the plasma membrane, cytoplasm, and gaps between adjacent damaged PTECs. This process enables mitochondria to repair damaged PTEC *in vivo* and further promotes the recovery and reconstruction of the overall structure of the renal tubules [47]. Studies have confirmed the safety of autologous BM-MSC transplantation *via* the venous or dorsal pancreatic artery route in the treatment of type 2 diabetes mellitus [128]. While the relationship between mitochondrial dysfunction and the onset or progression of metabolic diseases has been established, the therapeutic potential of MSC mitochondrial transfer remains largely untapped due to a lack of understanding of the mechanism. Nonetheless, there is significant therapeutic promise in this process, prompting further experimentation in later studies to elucidate the molecular and cellular mechanisms of MSC mitochondrial transfer and validate its efficacy as a foundation for future clinical trials.

### 4.2 Tissue Repair and Regeneration

#### 4.2.1 Enhancing Wound Healing and Tissue Regeneration

MSC mitochondrial transfer represents a promising therapeutic strategy for enhancing wound healing and tissue regeneration by improving cellular function, reducing oxidative stress, promoting angiogenesis, and modulating inflammation. Mitochondrial dysfunction plays a crucial role in the pathogenesis of related corneal and retinal diseases. In the pathogenesis of age-related macular degeneration, RPE utilizes glucose from photoreceptors to produce ATP *via* the glycolytic pathway due to mitochondrial dysfunction, resulting in apoptosis of photoreceptors and/or RPE cells. In the pathogenesis of diabetic retinopathy, mitochondrial dysfunction occurs in the retinal vascular system, which increases mitochondrial DNA damage and mitochondrial fragmentation, ultimately leading to apoptosis earlier than in retinal endothelial cells [86]. Thus, the key mechanism for achieving regeneration of retinal ganglion cells and corneal epithelial cells is intercellular mitochondrial transfer. Mitochondrial transfer plays an important role in the protection of ocular cells such as retinal ganglion cells (RGCs) and corneal epithelial cells [129]. MSC cells function to promote corneal wound healing and prevent corneal scar formation mainly through paracrine effects. In addition to this, MSCs can directly protect corneal epithelial cells (CECs) from Rotenone-induced mitochondrial damage by effectively transferring functional mitochondria. Notably, only MSCs with healthy mitochondrial function can effectively contribute to corneal wound recovery, and MSCs are more efficient at transferring mitochondria to CEC under stressful conditions through the NF-κB Signaling Pathway [57, 129]. Factors that damage the mitochondria of the corneal epithelium can lead to the development of dry eye [130]. The study showed that through TNTs, healthy human corneal epithelial cells can transfer mitochondria to damaged cells, restoring their energy requirements. Furthermore, utilizing the Rho kinase inhibitor Y-27632 can enhance the efficiency of mitochondrial transfer and improve the mitochondrial function of receptor CECs, thereby promoting the treatment and repair of dry eye [130]. Recent research demonstrated that implanting healthy MSCs cultivated on decellularized porcine corneal scaffolds into a rabbit model with ocular alkali injuries notably accelerated corneal wound healing [57]. Moreover, it was shown that RGC loss due to mitochondrial damage can be effectively prevented by iPSC-MSC transplanted into the vitreous by donating functional mitochondria to RGCs [129]. In a recent *in vivo* study, transplanted iPSC-MSC could protect RGCs from damage by transferring their mitochondria into recipient cells [131]. Thus, MSC mitochondrial transfer may provide a novel mechanism of action against corneal endothelial damage.

MSCs can stimulate or stabilize the bioenergetic potential of target cells, such as alveolar macrophages and alveolar epithelial cells, through mitochondrial transfer mechanisms, which facilitate the wound healing process. These mitochondrial transfer mechanisms are thought to achieve MSC therapy by modulating T lymphocyte proliferation, increasing epithelial proliferation, and inducing macrophage polarization to an M2 phenotype. Moreover, MSCs increase ATP concentration in recipient cells by transferring mitochondria to the alveolar epithelium, resulting in restoration of bioenergetics and alleviation of lung injury [48, 132]. MSCs have been shown to promote tissue repair and tissue homeostasis in a variety of lung injury models, including cigarette smoke-induced injury, experimental asthma, acute lung injury, heat shock, and bleomycin-mediated pulmonary fibrosis [37, 133]. Human iPSC-MSCs can restore lung injury induced by cigarette smoke in rats by donating healthy mitochondria to airway epithelial cells, which is superior to BM-MSCs [57, 134]. MSCs overexpressing Miro-1 factor transfer more mitochondria into mouse alveolar epithelial cells with ovalbumin- or fisetin-induced airway injury and trigger more efficient tissue repair compared with wild-type MSCs. Whereas MSCs knocked down for Miro-1 lost the ability to transfer mitochondria as well as the ability to repair [135]. In sepsis-induced ALI, MSCs mediate mitochondrial transfer *via* TNT, thereby improving the integrity of the pulmonary microvascular endothelial cells' barrier. The regulation of mitochondrial transcription factor A expression in MSC is critical in this process [136].

Moreover, the reparative capacity of MSC mitochondrial transfer has been observed in conditions such as stroke and myocardial infarction [59]. The introduction of MSC mitochondria can ameliorate symptoms associated with circulatory cardiomyopathy and mitigate neurological disorders like Alzheimer's disease by restoring aerobic respiration, enhancing mitochondrial membrane potential, and decreasing apoptosis [40]. In a mouse model of myocardial infarction, mitochondria-rich extracellular vesicles introduce mitochondria into cardiomyocytes, stimulate mitochondrial biogenesis, and enhance energy metabolism of failing myocardium, thus providing a new strategy for the treatment of cardiac diseases such as myocardial infarction and heart failure [137]. Additional investigations into the role of MSCs have indicated that mitochondrial transfers from MSCs also contribute to the reduction of apoptotic cells in rats with early spinal cord injury and diminish the area of glial scars and lesion space in advanced spinal cord injury cases [138]. Thus, MSC mitochondrial transfer plays a pivotal role in fostering wound healing and tissue regeneration.

#### 4.2.2 Modulating Immune Responses and Inflammation

Inflammatory diseases include a wide range of diseases characterized by excessive or uncontrolled inflammation, resulting in tissue damage and impaired function. Severe acute pancreatitis (SAP) is a prominent example, characterized by acute inflammation of the pancreas with significant systemic complications. Also in this category are respiratory infections such as chronic obstructive pulmonary disease (COPD) and acute respiratory distress syndrome (ARDS). However, MSC mitochondrial transfer has also shown promise in a variety of inflammatory and immune responses.

Most of the target cells of these diseases have impaired mitochondrial function, and MSCs can transfer mitochondria to damaged cells through mechanisms such as EV and TNT, thereby regulating immune response and inflammation. By providing functional mitochondria to injured cells, MSCs can restore cellular energy production, alter cellular metabolism, and affect inflammatory pathways. Hu *et al*. investigated the role of mitochondria from hypoxic MSCs in SAP. Their findings indicate that MSCs transfer mitochondria to pancreatic acinar cells *via* EVs, enhancing glycolytic flux and ATP production, which helps mitigate inflammation and supports cellular function under stress [139]. Alveolar macrophages (AM) are crucial in the innate immune response within the respiratory tract. MSCs have been shown to enhance AM phagocytic activity by transferring mitochondria through TNTs, improving pathogen clearance and inflammatory modulation [140]. Phinney *et al*. demonstrated that MSCs can transfer partially depolarized mitochondria to macrophages through EVs, enhancing oxidative phosphorylation and bioenergy, which supports macrophage function and inflammatory regulation [35]. However, in models of diabetic nephropathy, MSCs promote an anti-inflammatory macrophage phenotype through mitochondrial transfer, improving mitochondrial function, modifying cellular energy metabolism, and reducing inflammatory responses and kidney injury. The effects are mediated by mitochondrial biogenesis *via* PGC-1α and lysosomal autophagy through PGC-1α/TFEB [141-143].

The therapeutic potential of mesenchymal stem cell mitochondrial transfer extends to a variety of inflammation, and the above examples demonstrate that mesenchymal stem cell-based therapies can enhance inflammation control and tissue repair. This undoubtedly provides a very good direction for the field of histology and other fields, and provides strong evidence for the continued development of MSCs in this field. In addition, in the clinical aspect, MSCs also have a very good therapeutic effect on Graft versus host disease (GvHD) patients through mitochondrial transfer. Clinical studies have shown that long-term treatment of MSCs improves survival in patients with steroid-resistant acute GvHD [144]. This has brought great benefits to many patients with GvHD, especially special types of patients, and also laid a good foundation for the clinical application of MSCs in the treatment of immune response or inflammation.

Although there is substantial evidence that MSCs have a good therapeutic effect on the regulation of immune response and inflammation, most experiments are limited, and a detailed understanding of how MSC mitochondrial transfer regulates immune response and inflammation is still needed. Further prospective studies with sufficient certainty are needed to confirm efficacy and determine the role of mesenchymal stem cell therapy in the treatment of these diseases.

## 5 CHALLENGES AND CONSIDERATIONS

### 5.1 Optimization of Mitochondria Transfer Efficiency

Currently, mitochondrial transfer serves as an effective means of repairing damaged cells [12]. Research has revealed that various cell types, including fibroblasts and somatic cells, are capable of transferring mitochondria. However, mitochondrial transfer by MSCs offers distinct advantages, as it is more efficient, rapid, and cost-effective, allowing for the replacement of damaged mitochondria within cells, leading to better symptom reduction [12]. The majority of mitochondria are transferred unidirectionally from healthy cells to those with impaired mitochondrial function [145]. Using healthy cells for mitochondrial transfer enables efficient and precise delivery and function, thereby enhancing the efficiency of the transfer process [145]. Mitochondrial transfer between cells primarily occurs through TNTs [12]. The expression of connexins plays a crucial role in regulating mitochondrial transfer. For instance, overexpression of Miro1 in MSCs enhances the transfer of mitochondria from MSCs to damaged cells through TNTs [23]. Studies conducted by Islam *et al*. and Yao, Y. *et al* revealed the involvement of CX43 in the connection between MSCs and damaged cells [22, 36]. For mitochondrial transfer to occur, it is imperative to have the presence of CX43 expression along with GJC capability and form TNTs for transfer [21, 36]. Additionally, the extracellular environment also influences mitochondrial transfer. MSCs treated with N-acetyl-L-cysteine and L-ascorbic acid 2-phosphate showed improvements in mitochondrial quality, increased formation of TNT, and enhanced capability for mitochondrial transfer [146]. A greater MSC seeding density promotes the transfer of mitochondria [147]. In cell therapy, substantial quantities of mitochondria are necessary to be transferred, but current technology is constrained by low efficiency [148]. Good efficiency of transfer relies on the concentration of mitochondria. The integration of droplet microfluidics and co-culture techniques is employed to accomplish quantitative transfer. Moreover, with an increase in transfer duration, there is an increase in the number of mitochondria transferred, facilitating efficient and high-throughput mitochondrial transfer [148]. Several techniques are available to enhance mitochondrial transfer efficiency, such as centrifugation and heat shock during incubation, while others involve magnetic force-mediated transfer or Mitopunch technology, offering advantages in terms of speed and high throughput [21].

### 5.2 Quality Control and Safety Concerns

While numerous literatures indicate promising outcomes of mitochondrial transfer in treating various diseases, safety issues also exist. Transferring mitochondria from different individuals may trigger immune rejection and pose risks of mitochondrial mutation or heterogeneity, which could have serious consequences [15]. Several studies have indicated that allogeneic mitochondria might provoke inflammatory reactions [149]. The function and biogenesis of mitochondria rely on both the nuclear genome and the mitochondrial genome. Following mitochondrial transfer, alterations in nuclear-encoded proteins may lead to abnormal mitochondrial function [150]. Mitochondrial transfer mediated by EVs and TNTs may result in damaged or dysfunctional mitochondria due to stress incurred during the transfer process. In EV-mediated mitochondrial transfer, damaged mitochondria may be transferred, thereby affecting the metabolism and life of the recipient cells and potentially leading to adverse effects [15]. Excessive mitochondrial transfer intensity can pose safety concerns, including the potential for excessive GJC or hemichannel opening, which may disrupt cellular homeostasis and trigger cell death. Increased mitochondrial density signifies elevated ROS levels, subsequently resulting in heightened cytochrome C production and ultimately cell death. Additionally, excessive ATP may also be produced beyond the body's requirements, leading to adverse reactions [21]. The compatibility of transferred mitochondria with the nuclear DNA of the recipient cell is a crucial consideration. Fusion capability between internal and external mitochondria is another important factor to assess. Potential effects on recipient cell autophagy and mitochondrial function also require careful examination. Furthermore, the fate of exogenous mitochondria, whether they will be subjected to autophagy by the recipient cells, is a pivotal issue in mitochondrial transfer studies.

Worse still, mitochondrial transfer may also cause mitochondrial diseases [150]. Mitochondria transfer can exert negative effects on cancer cells and potentially cause tumor occurrence, recurrence, and drug resistance. MSCs exhibit altruistic behaviours, as they may strengthen the drug resistance of cancer cells and reduce therapeutic efficacy by transferring their mitochondria to cancer cells [12, 13, 15]. While cell-to-cell fusion offers a means to transfer mitochondria with intact structures and functions, fusion between healthy cells and cancer cells may result in the transmission of oncogenes or cancer-promoting agents, potentially facilitating the spread of cancer [15]. Numerous studies indicate that aside from targeting mitochondria to damaged cells, they may also be engulfed by other cells, leading to unnecessary cellular activation and undermining bodily stability [21]. The process of mitochondrial transfer poses challenges, with technical factors significantly influencing the success rate and associated complications. At present, mitochondrial transfer has shown safety in animal trials; nevertheless, the absence of human trial data remains a significant gap. Extrapolating results from animal experiments to humans entails limitations and potential safety risks, underscoring the importance of conducting more rigorous testing and monitoring in patients. Consequently, further research is essential to address these safety concerns and ensure the safety and efficacy of mitochondrial transfer as a treatment modality.

### 5.3 Immunogenicity and Host Response

**Fig. (3):**
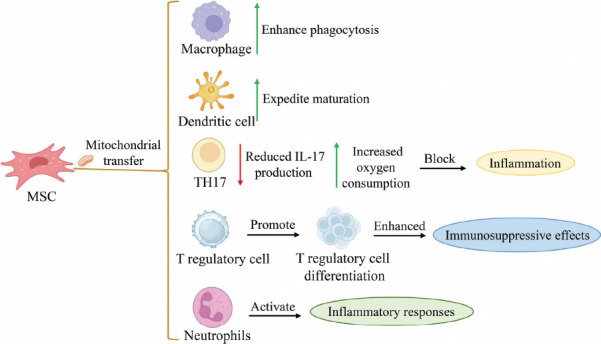
Immunogenicity and host response of mesenchymal stem cell (MSC) mitochondrial transfer. MSCs transfer mitochondria to immune cells, influencing their metabolism, differentiation, and immune response, thus promoting immune homeostasis restoration. Macrophages acquire healthy mitochondria from MSCs, enhancing phagocytosis. Dendritic cells recognize mitochondria and mitochondrial products, accelerating their maturation. Pathogenic Th17 cells, after receiving mitochondria from MSCs, exhibit altered responses, with increased oxygen consumption, reduced IL-17 production, and inhibited inflammatory function. Mitochondrial transfer stimulates T cell activation and regulatory T cell differentiation, leading to increased numbers of regulatory T cells and enhanced immunosuppressive effects. Additionally, intravenously injected outer mitochondria combine with mouse neutrophils, promoting neutrophil adherence to blood vessels and activating neutrophilic and inflammatory responses.

MSC mitochondrial transfer affects various cellular activities in recipient cells, including proliferation, differentiation, metabolism, inflammatory response, cell senescence, and cellular stress *etc* (Fig. **3**). The transfer of mitochondria from MSCs to other cells emerges as a potential strategy for modulating immune responses and addressing immune-related disorders [53]. Immune cells specifically recognize mitochondria and mitochondrial products (such as mtDNA), resulting in functional changes such as altering macrophage paracrine secretion or expediting dendritic cell maturation [49]. MSCs transfer mitochondria to immune cells, conveying signals that promote immune homeostasis restoration, influencing their metabolism, differentiation, and immune response [11, 45]. Alveolar macrophages will enhance phagocytic abilities after receiving mitochondria from MSCs, and exogenous mitochondria can facilitate macrophage differentiation into the anti-inflammatory phenotype [51, 53]. In acute respiratory distress syndrome, the acquisition of healthy mitochondria from MSCs by macrophages leads to enhanced phagocytosis [140]. In co-culture experiments, it was observed that after receiving mitochondria from MSCs, pathogenic helper Th17 cells experienced altered responses and functions, with increased oxygen consumption, reduced IL-17 production, and blocked inflammatory function [11, 53]. Mitochondrial transfer stimulates T cell activation and promotes the differentiation of regulatory T cells, resulting in increased numbers of regulatory T cells and enhanced immunosuppressive effects [151]. In a mouse experiment conducted *in vivo* by Boudreau *et al*., it was observed that intravenously injected outer mitochondria combined with mouse neutrophils led to neutrophil adherence to the blood vessel wall and activation of neutrophilic and inflammatory responses [152]. While mitochondrial transfer from MSCs profoundly affects immune cells, the precise mechanism through which mitochondrial transfer triggers host immune responses remains unclear, and further investigation and exploration are very necessary [51].

After receiving transferred mitochondria, recipient cells will undergo a series of responses, which will lead the damaged cells to exhibit enhanced resistance to oxidative stress damage [49]. However, mitochondrial transfer may cause rejection in recipient cells. In animal models, it was found that autologous mitochondrial transfer (except for congenital mitochondrial diseases) did not lead to inflammation-sensitive markers (TNF-α, IL-6, and C-reactive protein) and did not produce mitochondrial antibodies. These findings suggest that autologous mitochondrial transfer (except for congenital mitochondrial diseases) may prevent rejection and inflammation [153]. However, concerning allogeneic mitochondrial transfer, several studies have indicated that it does not provoke alloreactivity and recognition of DAMPs, with some studies suggesting that it may also provoke inflammatory responses [154]. In an *in vitro* experiment, allogeneic mitochondria were observed to activate human endothelial cells, leading to elevated levels of adhesion molecules, inflammatory cytokines, and chemokines [155]. Therefore, further research is required to elucidate the immune response outcomes of MSC mitochondrial transfer.

### 5.4 Long-term Stability and Persistence of Transferred Mitochondria

The effectiveness and feasibility of mitochondrial transfer in treating various diseases have been established through numerous literature studies. Therefore, it is imperative to investigate the long-term stability and persistence of transferred mitochondria. After transferring healthy mitochondria, MSCs regulate the replication of mtDNA in recipient cells and influence other mitochondria to maintain endogenous mitochondrial homeostasis. In patients with mitochondrial diseases, MSCs can be obtained from the patients themselves, allowing the healthy mitochondria generated by MSCs to be transferred to damaged tissues for specific treatment, thereby reducing the risk of immune rejection associated with transferring mitochondria from different individuals [15]. During mitochondrial transfer, using healthy cells to offer mitochondria avoids issues such as untargeted delivery and inactivation in the extracellular environment [145]. Targeted transfer can prevent potential immune responses of exogenous mitochondria to healthy cells and ensure the sustained and lasting effectiveness of mitochondrial transfer [145]. Besides, Ca^2+^ exerts a significant influence on mitochondrial homeostasis, with high Ca^2+^ levels being detrimental to mitochondrial integrity [145, 156]. Therefore, the ability of mitochondria to maintain their structural and functional integrity during transfer in an extracellular environment with elevated Ca^2+^ levels remains a crucial consideration for achieving stable mitochondrial transfer [145, 156].

The duration of mitochondrial retention in recipient cells is positively correlated with the therapeutic efficacy of mitochondrial transfer. Extending the retention period can eliminate the need for repeated administrations and promote a sustained and stable effect [21]. Picone *et al*. discovered that utilizing synaptosomes as carriers for mitochondrial transfer enables the preservation of mitochondria for an extended duration and even allows for cryopreservation, which provides an idea for achieving persistent mitochondrial transfer [21, 157, 158]. For the retention of exogenous mtDNA following mitochondrial transfer into recipient cells, Sercel, A.J., *et al*. have revealed that exogenous mtDNA can be permanently retained within ρ0 cells (mtDNA-deficient cells) by co-incubating mitochondria with recipient cells [159]. In mitochondrial transfer utilizing the MitoPunch device, Dawson, E.R., *et al*. have indicated that exogenous mtDNA replaced the endogenous mutated mtDNA in ρ0 recipient cells and remained permanently, enabling the exogenous mitochondria to exert a prolonged and stable influence on improving cellular status [160].

## 6 CURRENT RESEARCH AND FUTURE DIRECTIONS

Since their discovery, MSCs have attracted considerable attention in modern medicine for their capacity to replace damaged tissues and modulate immune responses [161]. Notably, MSCs have shown promising results in animal models and clinical studies related to various hepatic conditions such as cirrhosis and liver failure [162]. MSC mitochondrial transfer holds the potential to repair or even replace dysfunctional mitochondria within hepatocytes, thus aiding in the restoration of normal liver function. The primary approach involves leveraging the multipotent differentiation ability of MSCs to prompt their transformation into hepatocytes. Subsequently, the mitochondria within MSCs are transferred to damaged hepatocytes or alveoli *via* TNT and MVs. It's recognized that during reperfusion, mitochondria within cells generate significant amounts of ROS due to prolonged ischemia, resulting in impaired mitochondrial function. Thus, the treatment objective could focus on restoring mitochondrial function to alleviate ROS production. Gollihue *et al*. conducted a study where they transferred healthy mitochondria into damaged cells to replace dysfunctional ones, leading to a reduction in ROS production and restoration of normal mitochondrial function [163]. In an experimental model of ischemic heart disease, healthy mitochondria were observed to transfer from MSCs to cardiomyocytes with impaired mitochondrial function *via* TNTs, resulting in a decreased number of apoptotic cells [164]. For pulmonary diseases, MSC mitochondrial transfer has been observed to rescue damaged pulmonary cells from mitochondrial dysfunction [36, 122, 165-168]. MSC mitochondrial transfer serves to replace dysfunctional mitochondria in damaged cells, facilitating the restoration of their normal cellular functions [36, 137]. However, further experiments are required to delve deeper into its specific defensive mechanisms.

Since the discovery of mitochondrial transfer as a potential treatment for mitochondrial diseases, this technology has garnered significant attention from researchers. It is now recognized for its effectiveness in addressing various conditions including degenerative neurological diseases [149, 169], visual system disorders [170, 171] (specifically in repairing retinal ganglion cells), respiratory ailments [168], immune system disorders [171], and cardiovascular issues [122, 164, 172]. Horizontal transfer of mitochondria from donor cells to recipient cells afflicted with mitochondrial dysfunction [173]. Studies have revealed that mitochondria can spontaneously transfer from healthy donor cells (often MSCs) to recipient cells with mitochondrial dysfunction *in vivo*. However, due to the tropism of MSCs toward cancer cells [174], they become entrapped by cancer cells during mitochondrial transfer. Subsequently, the mitochondria are engulfed by cancer cells, interrupting the mitochondrial transfer pathway. This not only triggers carcinogenesis but also facilitates cancer metastasis [15, 60, 174, 175].

Cancer cells, characterized by their high proliferative capacity, exhibit uncontrolled growth not restrained by cell size or environmental factors. It is known that cancer cells acquire ATP not through glycolysis but *via* oxidative phosphorylation [176]. In a phenomenon where cancer cells exploit TNT for mitochondrial transfer with MSCs, mitochondria become co-opted by cancer cells. This event leads to an increase in mitochondrial respiration, consequently facilitating the influx of dihydroorotate dehydrogenase (DHODH) into cells. DHODH is a critical component for pyrimidine synthesis, thereby enhancing the ability of cancer cells to engage in oxidative phosphorylation. Consequently, some researchers propose DHODH as a potential therapeutic target for oxidative phosphorylation-dependent cancers [177]. Moreover, there's a school of thought among scholars suggesting that the tumor-affinitive properties of MSCs could be leveraged to transport anti-cancer drugs directly to tumor sites [174].

Numerous studies have underscored the association between mitochondrial dysfunction and various diseases. Mitochondrial transfer has emerged as a promising therapeutic approach, wherein functional mitochondria are transferred into cells with impaired mitochondrial function to replace damaged organelles. The concept of mitochondrial transfer between cells was first posited in 2005 [178]. Subsequently, Spees *et al*. discovered the process of mitochondrial transfer from healthy MSCs to mammals exhibiting mitochondrial dysfunction the following year [24]. This groundbreaking revelation confirmed the spontaneous transfer of mitochondria between cells, catapulting mitochondrial transfer to the forefront of regenerative medicine. To date, mitochondrial transplantation has demonstrated its unique efficacy in treating numerous diseases [179, 180]. Mitochondria can be obtained from various sources, including allogeneic cells or cells cultured *in vitro*, such as MSC mitochondria derived from induced pluripotent stem cells (iPSCs). iPSCs represent another cell type with robust differentiation potential, capable of being directed to differentiate into a wide array of cell types (*e.g*., MSCs, skin cells, *etc*.). However, in comparison to adult tissue-derived MSCs, iPSC-MSCs offer advantages such as an inexhaustible supply and readily solvable issues regarding source and quantity [51].

Moreover, iPSC-MSCs exhibit superior efficiency in repairing functional mitochondria and addressing cardiopulmonary injuries compared to adult bone marrow-derived MSCs [168]. iPSCs can be harvested from the recipient site where cells with impaired mitochondrial function reside, differentiated into healthy MSCs, and then utilized to transfer mitochondria to cells with impaired mitochondria. This approach resolves the rejection issues associated with *in vitro* mitochondrial transplantation [51].

## CONCLUSION

Since the discovery that MSC mitochondrial transfer can occur between cells, the world has focused on this discovery. The application of MSC mitochondrial transfer in diseases in different fields has been continuously updated. In particular, gratifying progress has been made in the treatment of diseases related to mitochondrial functional damage, such as liver disease, lung disease, ischemia-reperfusion injury, and cancer *etc*. In animal disease models, researchers have discovered the mechanism of MSC mitochondrial transfer, the signals that trigger mitochondrial transfer, and how MSCs regulate mitochondrial entry, receptor cells, and function. Unfortunately, the complete mechanism of inducing mitochondrial transfer, which signals trigger MSC mitochondrial transfer, whether the transfer of MSC mitochondria works together with other mechanisms, and the role of transferred mitochondria in host cells is still unclear. Despite promising preclinical outcomes, several barriers impede clinical translation, including variability in transfer efficiency, potential oncogenic risks due to bidirectional mitochondrial exchange, and unresolved immunogenicity concerns in allogeneic settings. For the successful initiation of clinical trials, it is essential to delve deeper into understanding the mechanism of MSC mitochondrial transfer and assess the feasibility of the technology. Additionally, further research is required to optimize the method of mitochondrial transfer, focusing on enhancing and refining the transfer process to maximize its effectiveness within host cells. Future interdisciplinary efforts integrating bioengineering (*e.g*., microfluidics, MitoPunch technology) and mitochondrial genomics will be pivotal in optimizing targeted delivery and ensuring long-term functional integration of transferred mitochondria.

## Figures and Tables

**Table 1:** Functional implications of mitochondria transfer in MSCs.

**Functional Implications**	**MSCs from Different Tissue Sources**	**Main Profile**	**References**
Enhancement of oxidative phosphorylation	BM-MSCs	↑ATP, Oxygen consumption, Phagocytosis by human macrophages, Citrate synthase activity, Complex I, Cancer cell bioenergetics ↓Pro-inflammatory cytokines	[39, 44, 45, 48, 51]
Regulation of ROS	BM-MSCs, DP-MSCs, WJ-MSCs, AD-MSCs	↑Mitochondrial SOD enzyme, Bcl-2 protein, SGLT2, Megalin transporter, renal tubular function, ↓ROS, TGF-β	[47, 51]
Differentiation potential and lineage commitment	BM-MSCs	↑Osteogenic differentiation ↓Cardiomyocyte apoptosis or degeneration	[23, 46, 47, 69]
Proliferation and self-renewal capacity	WJ-MSCs; BMSCs; CA-MSCs	↑Oxidative phosphorylation; Phagocytic activity of phagocytes; Biological functions; FLS proliferative capacity; Pro-angiogenic activity of MSC cells; Attachment-free proliferation; MtDNA; Aerobic viability; Respiratory function; OXPHOS; ATP; Bone defect healing; HUVECs; SCF; Occurrence of tumors; Chemoresistance; Tumor cell heterogeneity ↓Endothelial senescence; Neuronal apoptosis; Ischemic injury; Apoptotc tendon cells	[39, 60, 67, 84, 135, 140, 179, 180]

**Abbreviations:** BM-MSCs, bone marrow-derived mesenchymal stromal cells; ATP, adenosine triphosphate; ROS, reactive oxygen species; DP-MSCs, dental pulp- mesenchymal stromal cells; WJ-MSCs, Wharton's jellied-MSCs; AD-MSCs, adipose tissue- mesenchymal stromal cells; SOD, superoxide dismutase; CA-MSCs, cancer-associated mesenchymal stromal cells; FLS, fibroblasts-like synoviocytes; MtDNA, mitochondrial DNA; OXPHOS, oxidative phosphorylation; HUVECs, human umbilical vein endothelial cells; SCF, stem cell factor; ↑ indicates a significant increase compared to control (*p* < 0.05); ↓ indicates a significant decrease compared to control (*p* < 0.05).

**Table 2:** Therapeutic applications of MSC mitochondrial transfer.

**Therapeutic Applications**	**Application Aspects**	**Type of Diseases**	**Main Profile**	**References**
Mitochondrial dysfunction-related diseases	Neurodegenerative disorders	AD	↓Death of the recipient cells	[99]
	Cardiovascular diseases	Myocardial ischemic reperfusion injury	↑Mitochondrial membrane potential, Mitochondrial function, Aerobic respiration ↓Cell apoptosis, Myocardial fibrosis	[51, 114, 117]
		Anthracycline-induced cardiomyopathy	↑ATP ↓ROS, Cellular necrosis, DOX-induced myocardial fibrosis	[118, 122]
	Metabolic disorders	NAFLD	↑ATP ↓ROS	[126, 127]
Tissue repair and regeneration	Enhancing wound healing and tissue regeneration	AMD	↑Corneal wound healing ↓RGC loss	[57, 129, 131]
		ALI	↑Integrity of the PMVECs barrier, ATP, Bioenergetics	[48, 132]
	Modulating immune responses and inflammation	SAP	↑Glycolytic flux , Glycolytic reserve capacity, ATP	[139]
		ARDS	↑Basal respiration rate and mitochondrial ATP turnover rate of monocyte‐derived macrophages	[140]
		ALI	↑ATP, Oxidative phosphorylation, Bioenergetics, Phagocytosis of Alveolar macrophages ↓Proinflammatory cytokines	[35, 36, 48]

**Abbreviations:** AD, Alzheimer's disease; NAFLD, non-alcoholic fatty liver disease; AMD, age-related macular degeneration; ALI, acute lung injury; SAP, severe acute pancteatitis; ARDS, acute respiratory distress syndrome; PMVEC, pulmonary microvascular endothelial cell; ↑ indicates a significant increase compared to control (*p* < 0.05); ↓ indicates a significant decrease compared to control (*p* < 0.05).

## LIST OF ABBREVIATIONS

AD: Alzheimer's Disease
AD-MSCs: Adipose Tissue-derived Mesenchymal Stem Cells
ALI: Acute Lung Injury
ALS: Amyotrophic Lateral Sclerosis
AM: Alveolar Macrophages
AMD: Age-Related Macular Degeneration
ARDS: Acute Respiratory Distress Syndrome
ATP: Adenosine Triphosphate
Aβ: Amyloid β Protein
BM-MSCs: Bone Marrow-derived Mesenchymal Stem Cells
CECs: Corneal Epithelial Cells
COPD: Chronic Obstructive Pulmonary Disease
CVD: Cardiovascular Diseases
CX43: Connexin 43
DAMPs: Damage-associated Molecular Patterns
DCs: Dendritic Cells
DN: Diabetic Nephropathy
DOX: Doxorubicin
DP-MSCs: Dental Pulp-derived Mesenchymal Stem Cells
ETC: Electron Transport Chain
EVs: Extracellular Vesicles
GJCs: Gap Junction Channels
GvHD: Graft Versus Host Disease
HSPCs: Hematopoietic Stem and Progenitor Cells
HUVECs: Human Umbilical Vein Endothelial Cells
IFN-γ: Interferon-gamma
iPSC-MSCs: Induced Pluripotent Stem Cell-derived Mesenchymal Stem Cells
LIF: Leukaemia Inhibitory Factor
MDVs: Mitochondria-derived Vesicles
MSCs: Mesenchymal Stem Cells
mtDNA: Mitochondrial DNA
mtRNA: Mitochondrial RNA
MVs: Microvesicles
NAFLD: Non-alcoholic Fatty Liver Disease
NK: Natural Killer Cells
PD: Parkinson's Disease
PMVEC: Pulmonary Microvascular Endothelial Cell
PTECs: Proximal Tubular Epithelial Cells
RGCs: Retinal Ganglion Cells
ROS: Reactive Oxygen Species
RPE: Retinal Pigment Epithelium
SAP: Severe Acute Pancreatitis
SCF: Stem Cell Factor
TCA: Tricarboxylic Acid
TNF-α: Tumor Necrosis Factor-alpha
TNTs: Tunneling Nanotubes
WJ-MSCs: Wharton's Jelly-derived Mesenchymal Stem Cells
